# Inhibition of Thioredoxin Reductase by Santamarine Conferring Anticancer Effect in HeLa Cells

**DOI:** 10.3389/fmolb.2021.710676

**Published:** 2021-08-18

**Authors:** Junmin Zhang, Qianhe Xu, Hong-Ying Yang, Minghao Yang, Jianguo Fang, Kun Gao

**Affiliations:** School of Pharmacy, State Key Laboratory of Applied Organic Chemistry, and College of Chemistry and Chemical Engineering, Lanzhou University, Lanzhou, China

**Keywords:** thioredoxin reductase, reactive oxygen species, santamarine, oxidative stress, apoptosis, natural product

## Abstract

Natural products frequently have unique physiological activities and new action mechanisms due to their structural diversity and novelty, and are an important source for innovative drugs and lead compounds. We present herein that natural product santamarine targeted thioredoxin reductase (TrxR) to weaken its antioxidative function in cells, accompanied by accumulation of high levels of reactive oxygen species (ROS), and finally induced a new mechanism of tumor cell oxidative stress-mediated apoptosis. TrxR knockdown or overexpression cell lines were employed to further evaluate the cytotoxicity of santamarine regulated by TrxR, demonstrated that TrxR played a key role in the physiological effect of santamarine on cells. Santamarine targeting TrxR reveals its previously unrecognized mechanism of antitumor and provides a basis for the further development of santamarine as a potential cancer therapeutic agent.

## Introduction

Small molecule cancer therapeutics have perpetually played a crucial role in controlling tumor proliferation and progression, yet the primary challenge remains to discriminatively eradicate cancer cells with little or no toxicity to normal counterparts (Sun et al., 2015). Therefore, in this context, the development of small molecules that target cancer-related genomes is a particularly considerable alternative (Hahn et al., 2021). However, such small molecule drugs are normally only effective in specific patient groups due to the heterogeneity of tumors, and long-term application of tumors can usually circumvent its efficiency and become drug-resistant (Jin et al., 2019). Exploiting the biochemical changes in the tumor is another treatment approach (Hahn et al., 2021). It has been demonstrated biochemically that reactive oxygen species (ROS) levels are high in most cancer cells to maintain their malignant phenotypes and render tumor cells vulnerable to oxidative stress (Hayes et al., 2020). Considering this different redox state between normal cells and tumor cells, it is considered that the latter are more dependent on their ROS scavenging abilities. Therefore, exploring small molecules that target the antioxidant system to induce a further increase in oxidative stress will overwhelm this stress response in tumor cells, thereby leading to cell death (Gorrini et al., 2013; Zhang et al., 2021b).

The mammalian thioredoxin reductase (TrxR) enzymes are a vital redox regulator in cells and normally overexpress in tumor tissues to maintain tumor phenotypes and metastasis (Lincoln et al., 2003; Scalcon et al., 2018). Physiologically, TrxR protects normal cells from canceration, but if canceration still occurs, it also promotes cancer progression (Arner, 2017). The key function of TrxR is to sustain the essential redox regulating molecule thioredoxin (Trx) in its reduced state, thereby further interacting with a variety of downstream proteins and regulating their biological functions (Zhang et al., 2017b). Reduced Trx can regulate the activity of caspase and apoptosis signaling kinase 1 (ASK1) to affect cell apoptosis. Trx has been shown to directly inhibit cell apoptosis by catalyzing the S-nitrosylation of procaspase 3 and caspase 3 (Mitchell and Marletta, 2005). In addition, the reduced Trx binds to ASK1 to inhibit its activation, but the activation of ASK1 can lead to cell apoptosis (Liu and Min, 2002). These functions of Trx are directly controlled by its key physiological catalyst TrxR. Thus, targeting TrxR inhibition to develop cancer therapeutic agents has become an attractive strategy in cancer chemotherapy (Arner, 2017; Zhang et al., 2017b; Bian et al., 2019; Ghareeb and Metanis, 2020; Jovanovic et al., 2020; Jastrzab and Skrzydlewska, 2021).

Validation of the target and elucidation of the mechanism of the active molecules from natural products are the key to revealing the role of natural products. We discovered some small molecules targeting the redox biochemical characteristics of cancer cells to exert anticancer activity *via* regulating the redox state of cancer cells (Zhang et al., 2017c; Zhang et al., 2017d; Zhang et al., 2017e; Li et al., 2018; Zhang et al., 2018; Han et al., 2019; Li et al., 2019; Liu et al., 2019; Zhang et al., 2021c), and sesquiterpene lactone was one of them (Duan et al., 2016; Zhang et al., 2016). Such molecules work by reacting with functional groups available on proteins and enzymes (Babaei et al., 2018). As a paradigm, highly reactive Sec residue in the C-terminal penultimate of TrxR enzyme is a perfect functional group for sesquiterpene lactones (Duan et al., 2016; Zhang et al., 2016; Zhang et al., 2021c). We isolated santamarine, also called santamarin or douglanin, another sesquiterpene lactone containing α-methylene-γ-lactone moiety from the roots of *Inula racemose*. Santamarine has a variety of biological activities including anticancer (Ma et al., 2009; Mehmood et al., 2017; Wu et al., 2017), antibacterial (Coronado-Aceves et al., 2016), and antiinflammatory activities (Choi et al., 2012; Al-Attas et al., 2015; Jalal et al., 2020). It has been reported that santamarine stimulates oxidative stress to inhibit the activation of NF-kB or STAT3, and finally plays an antitumor effect (Mehmood et al., 2017; Wu et al., 2017). However, the molecular mechanism of santamarine-induced oxidative stress remains unrevealed. Since the structure of santamarine contains α-methylene-γ-lactone moiety, we thus speculated that santamarine likely interacts with TrxR to induce oxidative stress and eradicate cancer cells. As one of our continuing efforts to discover therapeutic molecules that regulate the cellular redox system, we report here that santamarine is a novel TrxR inhibitor. Santamarine selectively inhibits cancer cell proliferation and is connected with its targeting of intracellular TrxR. Knockdown or overexpression of the *TrxR* gene in cells aggravates or alleviates the cytotoxicity of santamarine to cells, respectively. Santamarine inhibits recombinant and cellular TrxR activity and promotes ROS accumulation to trigger oxidative stress-mediated apoptosis in HeLa cells. The revelation of the interaction between santamarine and TrxR unravels the mechanism by which santamarine induces oxidative stress-mediated apoptosis, and supports santamarine as a candidate anticancer therapeutic agent.

## Materials and Methods

### Reagents and Enzymes

MTT (3-(4, 5-dimethyl-2-thiazolyl)-2,5-diphenyl-2H-tetrazolium bromide), streptomycin, and penicillin were obtained from Amresco (Solon, OH). BSA, PMSF, and Na_3_VO_4_ were provided by Beyotime (Nantong, China). NADPH was obtained from Roche (Mannheim, Germany). The Annexin V-FITC and PI apoptosis assay kit was purchased from Zoman Biotech (Beijing, China). DMEM, puromycin, DMSO, G418, DCFH-DA, DHE, DTT, Hoechst 33342, Ac-DEVD-pNA, insulin, and CHAPS were products of Sigma-Aldrich (St. Louis, United States). FBS was a product of Sijiqing (Hangzhou, China). DTNB was a product of J&K Scientific (Beijing, China). All other reagents used were of analytical purity. The recombinant rat TrxR1 (WT TrxR1) was provided by Professor Jianqiang Xu (Dalian University of Technology, China). The *Escherichia coli* (*E. coli*) Trx was produced as described by our previous reference (Liu et al., 2014). The plasmids, *shTrxR1* and *shNT*, and the cell lines, HEK-*TrxR1* cells and HEK-*IRES* cells were provided by Prof. Constantinos Koumenis of the University of Pennsylvania, United States (Nalvarte et al., 2004; Javvadi et al., 2010). The establishment of HeLa-sh*TrxR1* cells and HeLa-sh*NT* cell lines refer to our previously published literature (Duan et al., 2014).

### Cell Cultures

Unless mentioned otherwise, the experimental cell lines HeLa, HepG2, HL-60, MDA-MB-231, BEAS-2B, and L02 were purchased from the Shanghai Institute of Biochemistry and Cell Biology. These cell lines were cultured under standard culture conditions of 37°C incubator, 5% CO_2_ atmosphere, and DMEM medium. HeLa-sh*NT* and HeLa-sh*TrxR1* cells were cultured under standard conditions and supplemented with 1 μg mL^−1^ puromycin. HEK-*IRES* and HEK-*TrxR1* cells were cultured under standard culture conditions, and 0.4 mg mL^−1^ G418 and 0.1 μM sodium selenite were supplemented for each cell subculture.

### Compound

Santamarine was isolated from the roots of *Inula racemosa* Hook. f. The purity analysis of santamarine was performed on Waters pump 1,525 and PDA 2998 series HPLC systems with reversed-phase C18 (4.6 mm × 150 mm, 5 μm) chromatographic columns at room temperature. The chemical characterization spectrum and HPLC purity analysis diagram of santamarine were shown in the Supporting Information (Supplementary Figures S1–S3). A 100 mM solution of santamarine was prepared in DMSO and stored at 20°C, and the final concentrations of DMSO are no more than 0.1% (V/V) in experiments unless otherwise noted.

Santamarine (Coronado-Aceves et al., 2016), white powder, ^1^H NMR (300, CDCl_3_) δ: 6.05 (1H, d, J = 3.1 Hz, H-13a), 5.39 (1H, d, J = 3.1 Hz, H-13b), 5.33 (1H, br. s, H-3), 3.93 (1H, t, J = 11.0 Hz, H-6β), 3.65 (1H, dd, J = 9.9, 6.5 Hz, H-1α), 1.82 (3H, br. s, H-15), 0.86 (3H, s, H-14). ^13^C NMR (75 Hz, CDCl3) δ: 75.3(d, C-1), 32.9 (t, C-2), 121.4 (d, C-3), 133.5 (s, C-4), 51.2 (d, C-5), 81.7 (d, C-6), 51.1 (d, C-7), 21.3 (t, C-8), 34.4 (t, C-9), 41.0 (s, C-10), 139.1 (s, C-11), 171.0 (s, C-12), 117.0 (t, C-13), 11.2 (q, C-14), 23.5 (q, C-15).

### Cell Viability Assay

HeLa, HepG2, HL-60, MDA-MB-231, BEAS-2B, and L02 cells (5 × 10^3^–1 × 10^4^/well) were added with the specified concentrations of the experimental drug and 0.1% DMSO, and grown in triplicate in 96-well plates at 37°C for 24 or 48 h. Cells were exposed to the specified periods, MTT reagent (5 μL/well, 5 mg mL^−1^) was then added and continued to incubate at 37°C for 4 h. Then 100 μL extraction buffer (5% isobutanol, 10% SDS, and 0.1% HCl) was employed to dissolve formazan crystals. Cell viability was calculated by reading the absorbance at 570 nm.

### Pure TrxR Activity Assay

The NADPH-reduced TrxR (final concentration was 160 nM) was incubated with 0, 5, 10, 25, or 50 μM santamarine in a 96-well plate at room temperature for 30, 45, or 60 min. The final incubation volume was 50 μL. The TE buffer (50 μL, pH 7.5) containing 200 μM NADPH and 2 mM DTNB was then added, and the absorbance was recorded at 412 nm during the initial 3 min (Zhang et al., 2021c). The relative percentage of the control group was used to express the activity of the pure TrxR activity.

### Molecular Docking Simulation

As we mentioned previously (Zhang et al., 2021c), the crystal structure of rat TrxR1 (PDB code 3EAN) was used in the docking study. The residue Sec498 in chain A was chosen and further prepared in the protein preparation wizard module as a reactive residue involving Michael addition. The docking simulation was performed with default parameters.

### TRFS-Green-Based Live-Cell Imaging

According to our previously established TRFS-green-based live-cell imaging assay (Zhang et al., 2014), HeLa cells (1 × 10^5^) were seeded in a 6-well plate, and cultured overnight. Subsequently, the HeLa cells were exposed to 0, 10, 25, or 50 μM santamarine and further cultured for 8 h, and then treated with TRFS-green (final concentration is 10 μM) for 4 h at 37°C in dark. The images were captured on a fluorescent microscope.

### Trx-Mediated Endpoint Insulin Reduction Assay

HeLa cells were exposed to 0, 10, 25, or 50 μM santamarine for 24 h, and harvested. The RIPA buffer and the Bradford procedure were employed to extract and quantify the total cellular proteins. Intracellular TrxR activity was measured according to our published procedures (Duan et al., 2014; Zhang et al., 2021c).

### ROS Production Assay

HeLa cells (1 × 10^5^) were plated into a 12-well plate and were cultured overnight. HeLa cells were exposed to 0, 10, 25, or 50 μM santamarine for indicated times, and subsequently incubated with DCFH-DA or DHE (each final concentration was 10 μM) for 30 min at 37°C in dark with a method as described previously (Zhang et al., 2021c).

### Cellular Total Thiols Assay

5, 5′-Dithiobis-2-nitrobenzoic acid (DTNB) assay for cellular total thiols. After treating HeLa cells with santamarine, collect the cells and lyse them with RIPA buffer for 30 min on ice, vortexing occasionally. The total thiol level was determined by DTNB titration according to the method we previously described (Zhang et al., 2016).

### Detection of Morphological Changes of Apoptotic Cells

The morphological changes of apoptotic cells were detected by the dye Hoechst 33342 as described previously (Zhang et al., 2021c). Briefly, HeLa cells (1 × 10^5^) were seeded in a 12-well plate, and cultured overnight. Subsequently, the HeLa cells were exposed to 0, 10, 25, or 50 μM santamarine for 24 h. Hoechst 33342 (5 μg mL^−1^) was added and continued to incubate for 30 min. The stained nuclei were photographed under a fluorescence microscope.

### Caspase 3 Activity Detection

HeLa cells were exposed to 0, 10, 25, or 50 μM santamarine for 24 h, and lysed with RIPA buffer. Bradford procedure was employed to quantify the total cellular protein contents. The total proteins (120 μg) from extracts were incubated with the protease activity assay mixtures (100 μL, including 10 mM DTT, 5% glycerol, 2 mM EDTA, 0.2 mM Ac-DEVD-pNA, and 0.1% CHAPS in 50 mM Hepes, pH 7.5) for 2 h at 37°C. The caspase 3 activity was assessed by detecting the absorbance at 405 nm as described previously (Zhang et al., 2021c).

### Detection of Apoptotic Cells

Annexin V-FITC/PI staining was used to detect the number of apoptotic cells (Zhang et al., 2021c). HeLa cells (1 × 10^5^) were seeded in a 6-well plate, and cultured overnight. Then, the HL-60 cells were exposed to 0, 10, 25, or 50 μM santamarine for 24 h. The cells were harvested and washed with PBS. According to Annexin V-FITC/PI dual staining apoptosis detection kit, the apoptotic cells after santamarine action were determined and analyzed by flow cytometry with Cell Quest software (BD Biosciences, USA).

### Statistics

The Students t-test is used to evaluate the statistical difference between the two sets of data, and a one-way ANOVA is used for multiple groups. All experimental values are expressed as the mean ± S.E. of three independent experiments. The value of *p* < 0.05 was regarded as a statistically significant standard.

## Results

### Santamarine Used in This Study Derives From *Inula racemosa*

**FIGURE 1:**
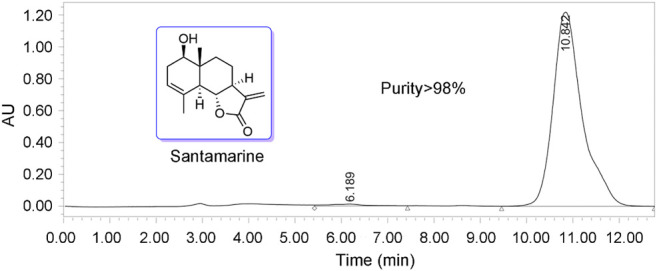
Structure of santamarine and analysis of purity (purity > 98%).

In our previous research, we presented multiple sesquiterpenoids isolated from plants in the *Asteraceae* family (Ma et al., 2013; Chen et al., 2014; Chen et al., 2015; Zheng et al., 2019). Among these sesquiterpenes, we found that sesquiterpene lactones exhibit excellent antitumor activity (Ma et al., 2013; Yang et al., 2016; Zhang et al., 2016; Zhang et al., 2021c). In the current study, santamarine (Figure 1), a sesquiterpene lactone isolated from *Inula racemosa*, was selected for mechanistic study in human cervical cancer cells. The chemical purity of santamarine was analyzed by HPLC, which suited the study of monomer compound activity (Figure 1). Santamarine was thus used to evaluate the proliferation inhibitory effect of multiple human tumor cells. Due to the presence of α, β unsaturated ketone on the lactone ring in the santamarine, human TrxR with a C-terminal active site Sec residue is speculated to be a potential target of the santamarine.

### Santamarine has Cancer-Selective Antiproliferative Activity

**FIGURE 2:**
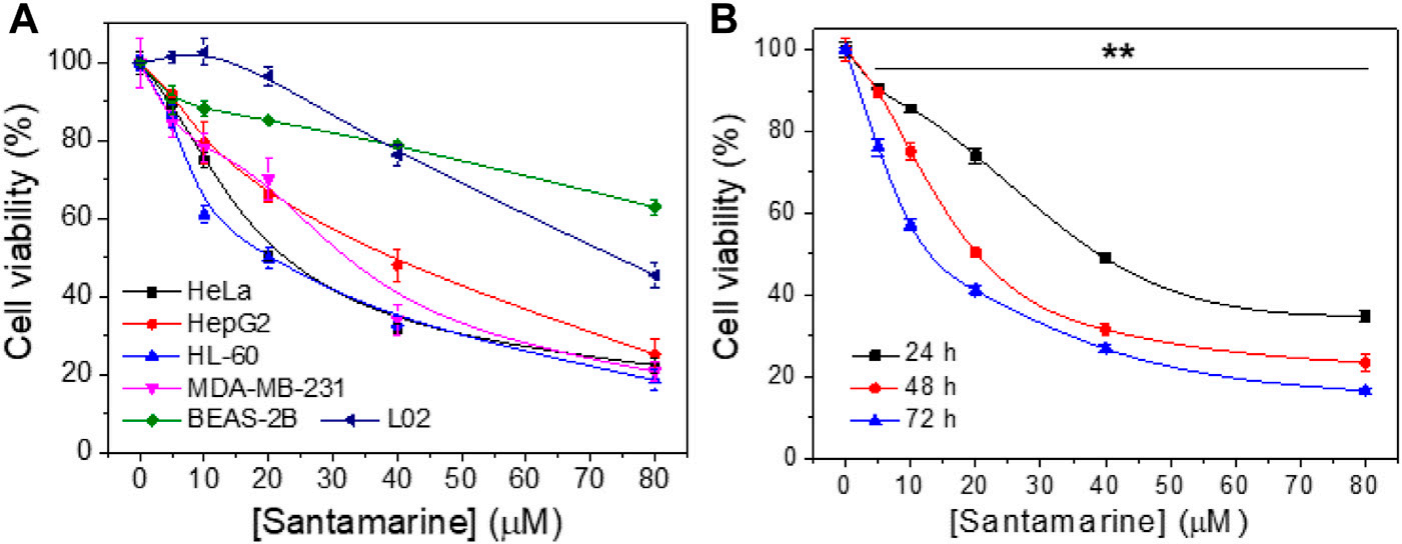
Santamarine harbors cancer-selective antiproliferative activity. **(A)** Cell viability of cancer cell lines (HeLa, HepG2, HL-60, and MDA-MB-231) and noncancerous cell lines (BEAS-2B and L02) after treatment with santamarine. The viability was detected by the MTT assay after cells were incubated with increasing concentrations of santamarine for 48 h **(B)** HeLa cell viability assays in 24, 48, and 72 h were carried out as in **(A)**, with a range of concentrations of santamarine. The results of each cell line were normalized to untreated controls. (*n* = 3 biological replicates, data are mean ± s. d. **p < 0.01 vs. the BEAS-2B group in **(A)**, and the control group in **(B)**.

First, santamarine was tested to determine its antiproliferative activity in a variety of human cancer cell lines or non-malignant cell lines as shown in Figure 2A. As described in the materials and methods, the cells were treated with increasing concentrations of santamarine for 48 h, and the MTT assay was employed to evaluate the cell proliferation. For each cell line, viability level was normalized to the proliferation of untreated cells. The results revealed that santamarine has a statistically significant, dose-dependent growth inhibition of all tested malignant cell lines HeLa, HepG2, HL-60 and MDA-MB-231, but has a much less inhibitory effect on the proliferation of nonmalignant cell lines BEAS-2B and L02 at our experimental concentrations (Figure 2A). Specifically, 40 μM santamarine inhibited the growth of HeLa cells at 48 h by more than 50%, whereas the inhibition rate for BEAS-2B was less than 20% (Figure 2A). In addition, santamarine showed a potent time-dependent antiproliferative effect on the cervical cancer cell line HeLa cells (Figure 2B). These results indicate that santamarine exhibits robust cell selectivity and is an ideal antitumor lead agent.

### Santamarine Inhibits TrxR in Cell-free Systems

**FIGURE 3:**
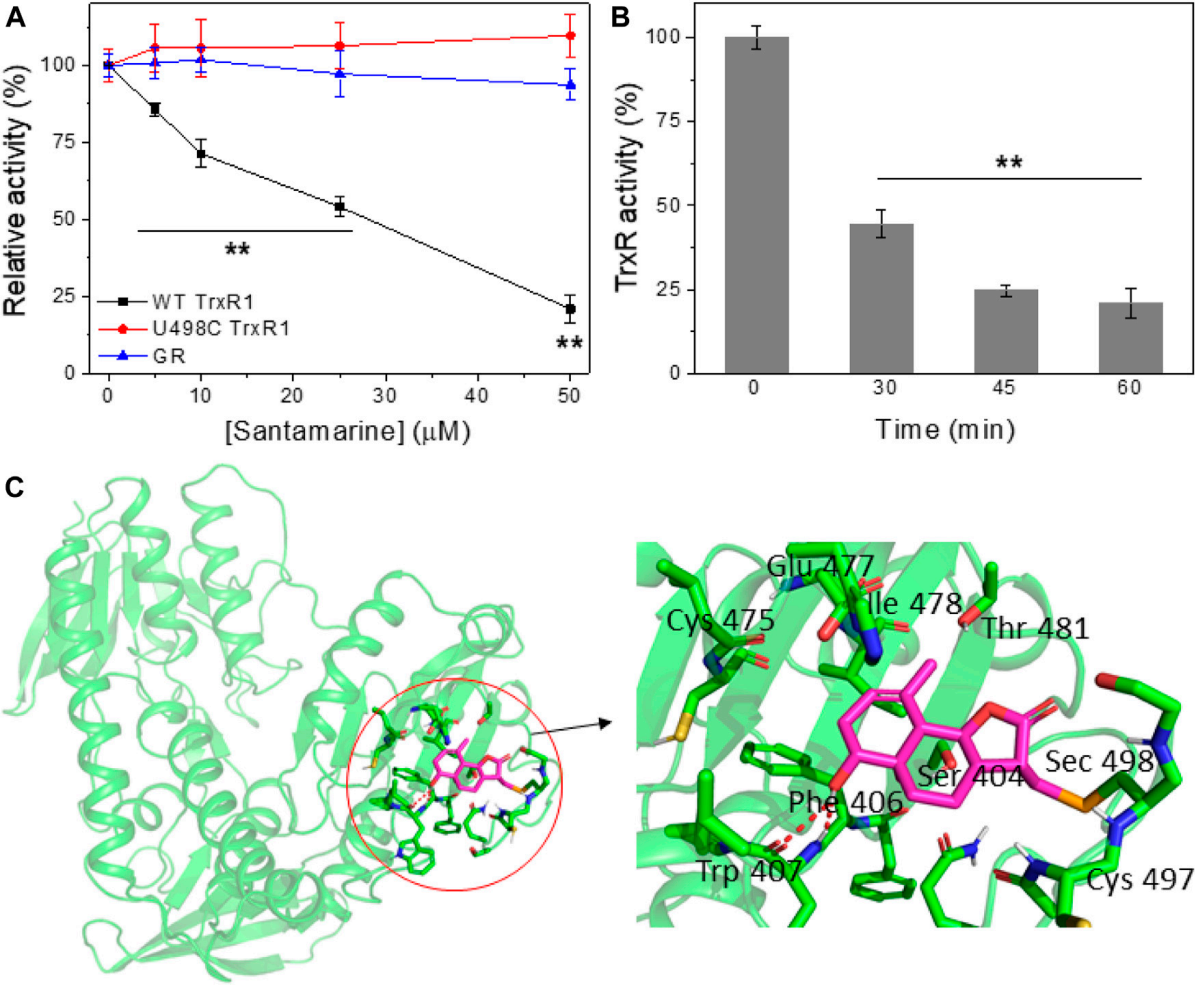
Santamarine is reactive with TrxR *in vitro*. **(A)** TrxR was inhibited by santamarine in a dose-dependent manner compared with the U498C TrxR and GR. The specified concentrations of santamarine were added to NADPH-reduced WT TrxR1, U498C TrxR1 and GR, and the mixed system was subsequently incubated at room temperature for 2 h. The enzyme activity was finally detected by DTNB reduction. **(B)** TrxR activity was inhibited by santamarine in a time-dependent manner. Santamarine (50 μM) and NADPH pre-reduced WT TrxR1 were incubated at room temperature for a specified time, and the activity was evaluated by DTNB method. **(C)** Molecular docking of santamarine and TrxR1 protein. The C-terminal position 498 of chain A of mouse TrxR1 was covalently docked with santamarine through the covalent docking protocol in the Schrödinger Suite 2015-1 program. The green cartoon indicates the monomer of TrxR1. The interaction between TrxR1 active site residues and santamarine is shown in orange and magenta sticks. The key residues around santamarine include Sec498, Cys497, Thr 481, Glu478, Ile477, Cys475, Trp407, Phe406, and Ser404. Data represent mean ± s. d. of three independent determinations. **p < 0.01 vs. the U498C TrxR and GR groups in **(A)**, and the control group in **(B)**.

Electrophilic Michael receptors can inhibit mammalian TrxR containing Sec residue (Gan et al., 2013). Since santamarine exhibits a robust cancer-selective antiproliferative activity, then we explore the mechanism of santamarine targeting TrxR as previously predicted. To study the possible inhibitory effect of santamarine on mammalian TrxR, we incubated santamarine at various concentrations with 160 nM NADPH-reduced recombinant rat TrxR for different times at room temperature. TrxR activity was then determined by the DTNB reduction assay. As shown in Figure 3 A and B, santamarine caused a dose- and time-dependent decrease in TrxR activity, respectively. Compared with U498C TrxR (the Sec498 was replaced by Cys) and glutathione reductase (GR, a homolog of TrxR), santamarine better inhibited WT TrxR due to the higher electrophiles of Sec compared to Cys, suggesting that the Sec active site of the enzyme was involved in the inhibition by santamarine in cell-free systems. We further confirmed the role of santamarine and Sec through molecular simulations. The residues and structure that form the binding site were shown in Figure 3C. The key residues around santamarine included Sec498, Cys497, Thr 481, Glu478, Ile477, Cys475, Trp407, Phe406, and Ser404. Thus, the proposed reaction mechanism for santamarine is to block the adjacent C-terminal active site residues Cys and Sec of TrxR, which is expected to effectively suppress TrxR activity. Generally, the inhibitory activity of TrxR is correlated with antitumor ability, indicating that TrxR is a potential molecular target of santamarine.

### Santamarine Inhibits Cellular TrxR Activity at Cytotoxic Concentrations

**FIGURE 4:**
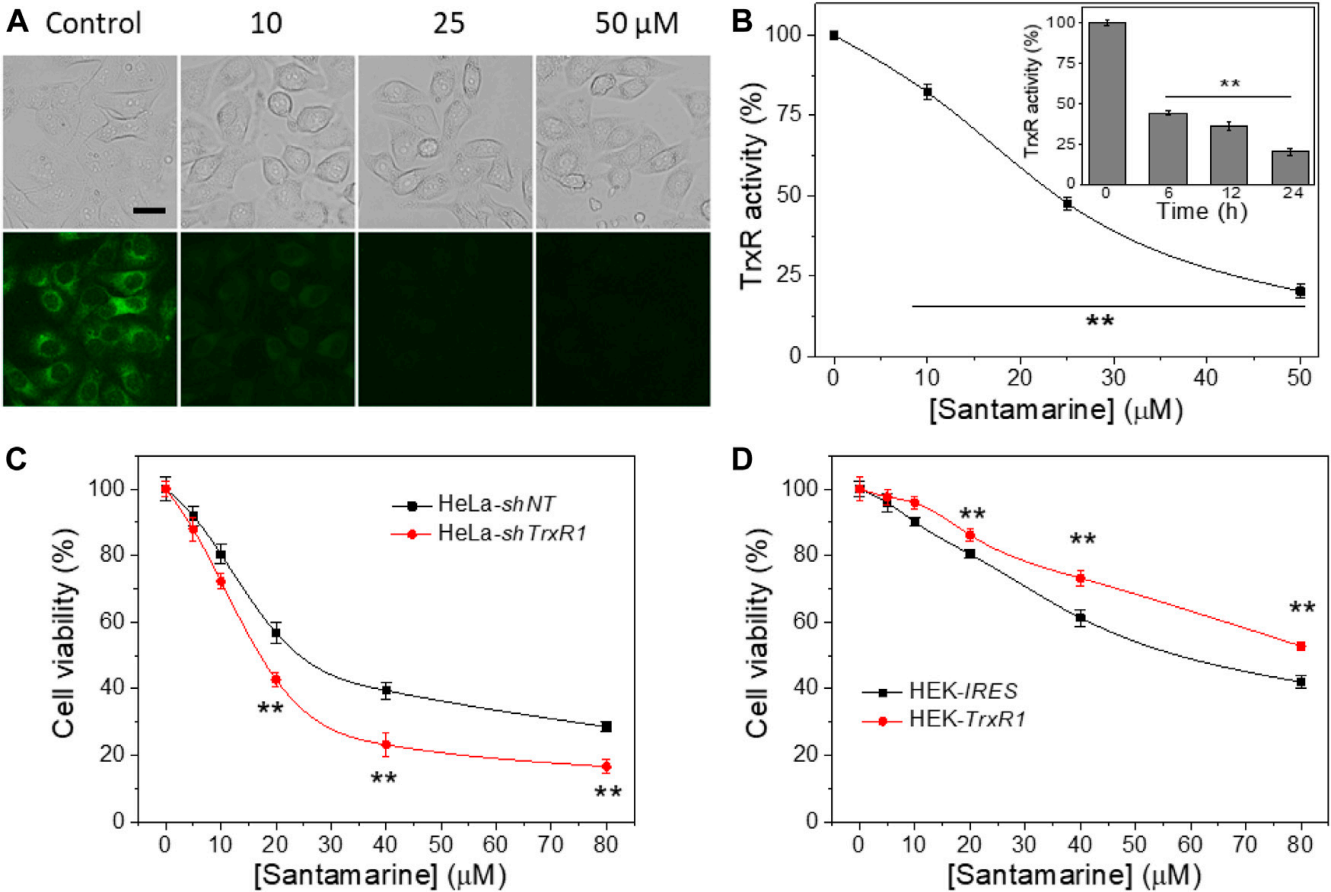
TrxR is a target for santamarine in cells. **(A)** Santamarine inhibited TrxR activity in live HeLa cells, as assessed by TRFS-green imaging. The top panel (bright field pictures) and the bottom panel (fluorescence pictures) were acquired by an inverted fluorescence microscope. The images represent three independent experiments performed in triplicate. Scale bar: 20 μm. **(B)** The effect of santamarine treatment on TrxR activity of HeLa cells was evaluated by the endpoint insulin reduction assay. HeLa cells were treated with different concentrations of santamarine for 24 h, and then TrxR activity in cell lysate was measured using an endpoint insulin reduction assay and expressed as a percentage of the DMSO-treated control. The inset in **(B)** reflects the time-dependent inhibition of TrxR by santamarine in HeLa cells. After the HeLa cells were treated with 50 μM santamarine for an indicated time point, the TrxR activity in cell lysate was also measured by the endpoint insulin reduction assay. **(C)** and **(D)** TrxR is indeed involved in the cellular process of santamarine. **(C)** Knockdown of TrxR1 sensitizes the cells to santamarine treatment. After 48 h of treatment of HeLa-*shNT* cells (control) and HeLa-*shTrxR1* cells (knockdown) with the indicated concentrations of santamarine, the cell viability was determined by MTT assay. **(D)** Overexpression of TrxR1 protects the cells from santamarine treatment. The HEK-*IRES* cells (control) and HEK-*TrxR1* (overexpression) cells were with the designed concentrations of santamarine for 48 h, and the cell viability was assessed by the MTT assay. All data are from three independent experiments in triplicate. **p < 0.01 versus the control group in **(B)**, between two cell lines in **(C)** and **(D)**.

The ability of santamarine to inhibit TrxR activity in human cervical cancer HeLa cells was tested. As described in the materials and methods, TRFS-green (a TrxR activity detection probe) imaging was firstly used to detect the activity of TrxR in cells treated with santamarine (Zhang et al., 2014). As the concentration of santamarine increased, the fluorescence intensity of the stained cells was reduced by TRFS-green staining. The cytotoxic concentration of santamarine almost fully inhibited TrxR in HeLa cells (Figure 4A). The endpoint insulin reduction assay was then employed to measure TrxR activity in santamarine-incubated cells. After treatment of HeLa cells with different concentrations of santamarine for 24 h, TrxR activity in the cell lysates decreased with increasing concentration of santamarine (Figure 4B), consistent with the observation from the live-cell imaging, further suggesting that santamarine inhibited the intracellular TrxR. Additionally, HeLa cells were treated with a higher cytotoxic concentration of santamarine (50 μM) at different time points, the TrxR activity in HeLa cells decreased in a time-dependent manner (insert in Figure 4B). The results indicate that the potential of santamarine to inhibit TrxR at cytotoxic concentrations probably contributes to its efficacy in suppressing cancer cell proliferation.

### TrxR is Involved in the Cellular Role of Santamarine

We evaluated the difference in the cytotoxicity of santamarine in *TrxR* gene knockdown and overexpression cell lines, further confirming the correlation between TrxR inhibition and cytotoxicity induced by santamarine. As shown in Figure 4C, santamarine was significantly more cytotoxic to HeLa-*shTrxR1*, a cell line that established stable knockdown of *TrxR1* gene by our lab previously developed (Duan et al., 2014; Duan et al., 2016), than non-targeted shRNA-transfected control cells (HeLa-*shNT* cells). We also compared the sensitivity of our previously acquired HEK cells stably overexpressing *TrxR1* gene (HEK-T*rxR1*) and cells stably transfected with the vector (HEK-*IRES*) to santamarine treatment (Duan et al., 2014; Duan et al., 2016). Interestingly, santamarine showed higher cytotoxicity to HEK-*IRES* cells than to HEK-*TrxR1* cells (Figure 4D). The response of santamarine to the cytotoxicity of TrxR knockdown or overexpression cell lines coherently suggests that TrxR is possibly involved in the cellular role of santamarine.

### Santamarine Induces ROS and Regulates the Cellular Stress Response

**FIGURE 5:**
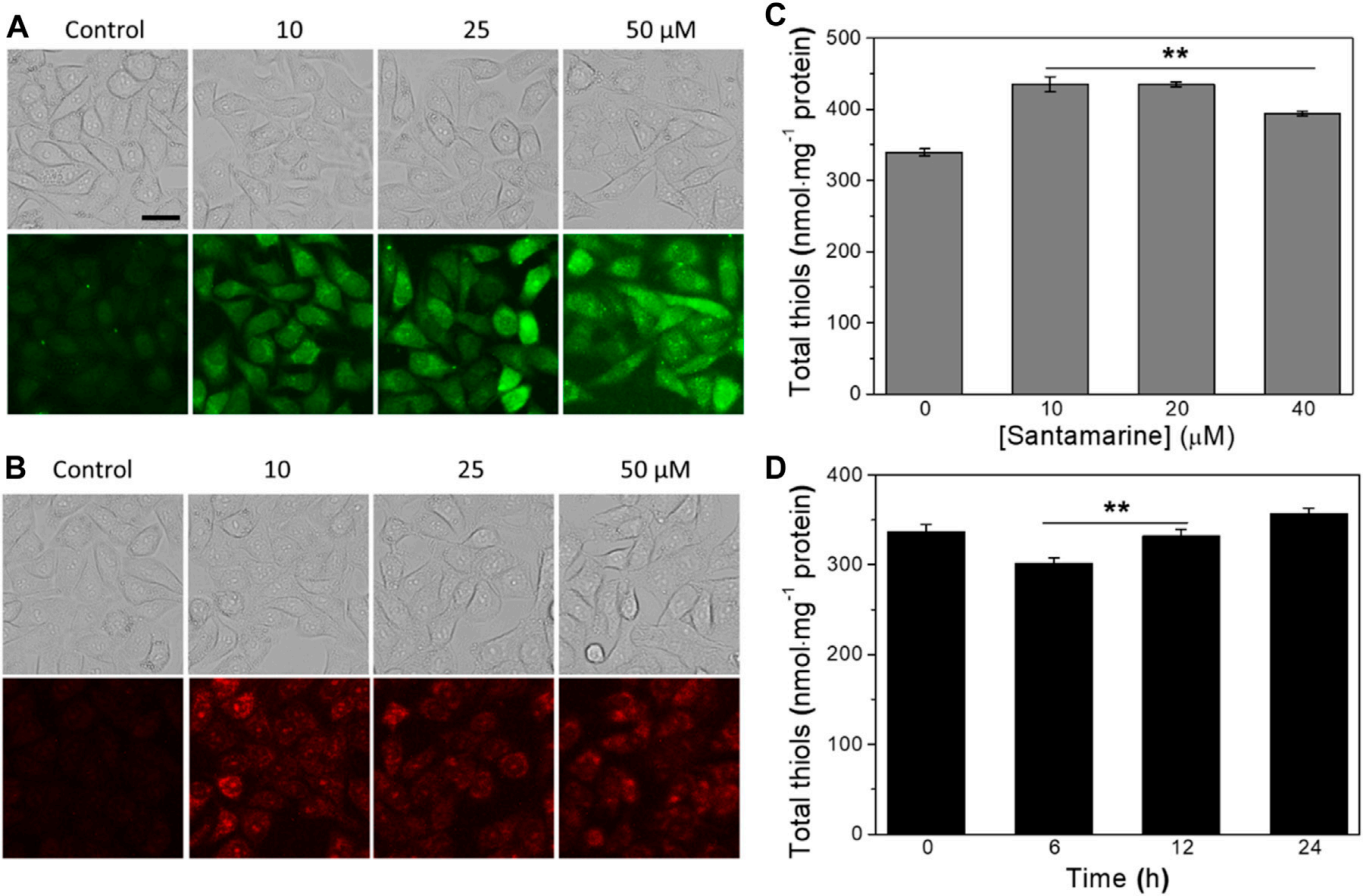
Santamarine induces ROS and regulates the cellular stress response. **(A)** The intracellular ROS accumulation was determined by DCFH-DA staining. **(B)** The intracellular superoxide anion accumulation was determined by DHE staining. After treating HeLa cells with vehicle (control) or santamarine at indicated concentrations for 6 h, they were stained with DCFH-DA (10 μM) and DHE (10 μM) for 30 min, respectively, and analyzed by fluorescence microscope to obtain phase contrast (top) and fluorescence (bottom) images. The images represent three independent experiments performed in triplicate. Scale bar = 20 μm. **(C)** The intracellular total thiol homeostasis responded to an increasing concentration of santamarine treatment. **(D)** The intracellular total thiol homeostasis responded to different times of a fixed concentration of santamarine treatment. After the increasing concentrations of santamarine (10, 20, or 40 μM) were exposed to treat HeLa cells for 24 h, or 50 μM of santamarine was exposed to treat HeLa cells for an indicated time (6, 12, or 24 h), the total thiol level in cell lysis was used DTNB titration determination. All data were from three independent experiments in triplicate. **p < 0.01 vs. the control group in **(C)**, the 24 h group in **(D)**.

The thioredoxin system maintains intracellular redox homeostasis by eliminating ROS (Zhang et al., 2021a). Multiple TrxR inhibitors can strongly enhance intracellular ROS levels, destroy intracellular redox homeostasis, and cause eventually tumor cell apoptosis (Cai et al., 2012; Zhang B. et al., 2017; Zhang et al., 2019; Arner, 2021; Chupakhin and Krasavin, 2021). Therefore, we checked whether santamarine induces ROS generation following TrxR inhibition. HeLa cells were treated with the indicated concentrations of santamarine for 2, 4, 6, 8, and 12 h, and the ROS-sensitive DCFH-DA dye was employed to measure intracellular ROS levels. Our results revealed that the intracellular ROS boosted dramatically after the cells were handled with santamarine for 6 h. Moreover, santamarine potently enhanced the ROS generation in HeLa cells in a concentration-dependent manner (Figure 5A). As such, the superoxide anion levels in HeLa cells treated with santamarine for 6 h were detected by the specific probe DHE. The santamarine-treated cells also arouse the fluorescence, suggesting the superoxide anion was generated (Figure 5B). These results together indicate that santamarine induces the accumulation of ROS in cells.

Given that the intracellular total thiol level has emerged as a marker of redox homeostasis (Baba and Bhatnagar, 2018; Hu et al., 2019), we then measured the cellular total thiol homeostasis following santamarine treatment. As shown in Figure 5C, the intracellular total thiols were provoked with the increasing concentrations of santamarine (10, 20, or 40 μM) treatment for 24 h (Figure 5C). As such, the cells were treated with santamarine at a fixed concentration (50 μM) for 6, 12, or 24 h. The results showed that the decrease of intracellular total thiols at 6 and 12 h was lower than that of the control and 24 h groups (Figure 5D). Because santamarine stimulated cells to produce more intracellular ROS accumulation at 6 h (Figures 5A,B), while the corresponding total thiols were slightly reduced to resist intracellular oxidative stress. However, when santamarine acted on cells for a long time, such as 24 h, the total thiols in the cells were higher than those in the control group. This is likely due to the activation of the ARE-Nrf2 cytoprotective pathway. It has been reported that molecules with an α, β-unsaturated ketone structure are generally effective Nrf2 activators (Peng et al., 2015; Peng et al., 2019; Osama et al., 2020). Altogether, santamarine stimulates ROS accumulation and disrupts the redox homeostasis in cells.

### Santamarine Effects on Cell Apoptosis

**FIGURE 6:**
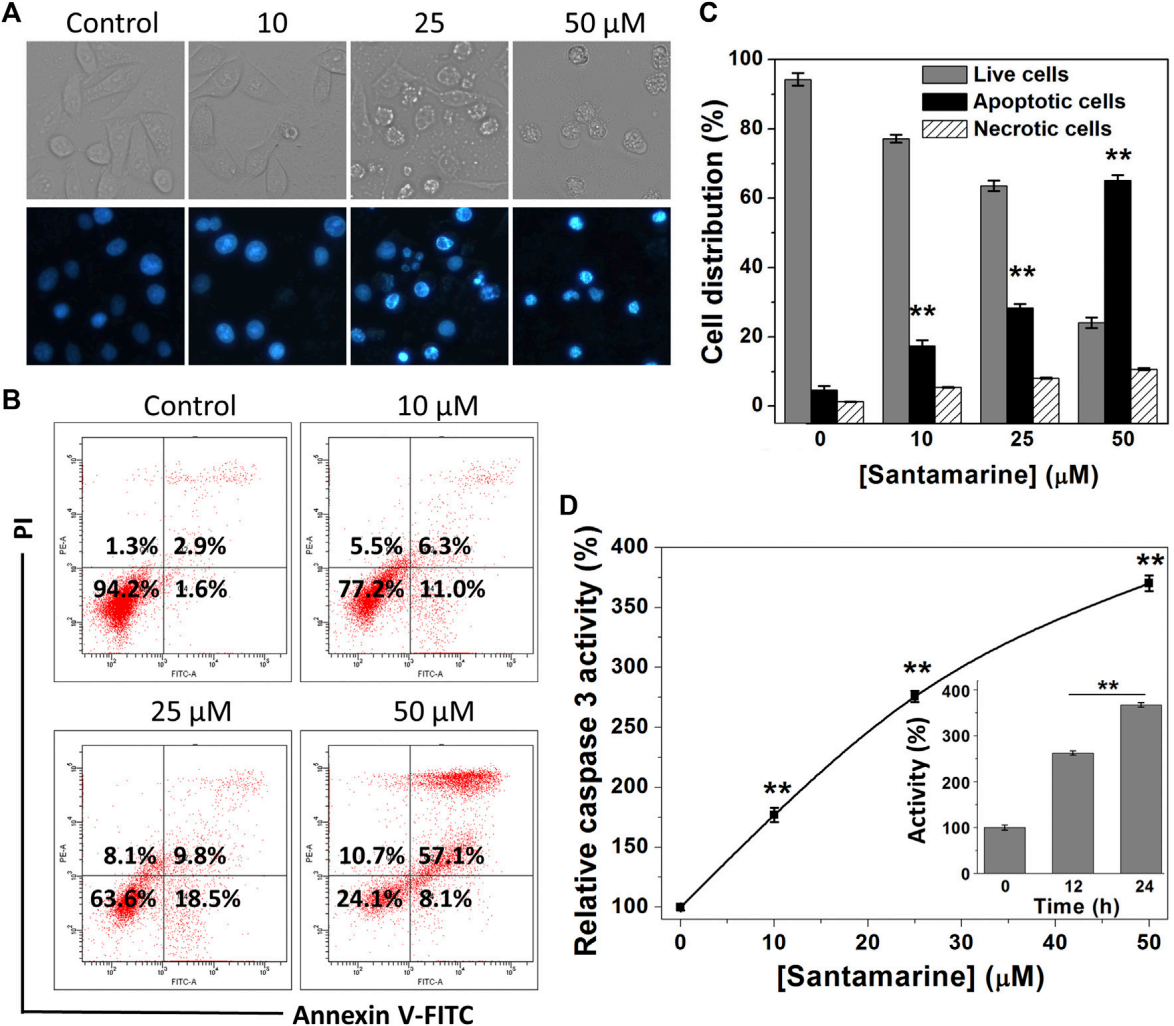
Santamarine effects on cell apoptosis. **(A)** HeLa cells were treated vehicle (control) and different concentrations of santamarine for 24 h, and the images of the cells stained with Hoechst 33342 solution were captured in representative phase contrast (top) and fluorescence (bottom). The images represent three independent experiments performed in triplicate. Scale bar = 20 μm. **(B)** HeLa cells were treated with the specified dose of santamarine for 24 h, and then analyzed for the Annexin V-FITC and PI staining by flow cytometry. **(C)** The average value and SD of live cells, apoptotic cells, and necrotic cells in three independent experiments stained with Annexin V-FITC and PI after 24 h of HeLa cells treated with santamarine. **(D)** Santamarine activated intracellular caspase 3 activity in a concentration and time-dependent manner. HeLa cells were incubated with santamarine at the specified concentrations for 24 h, or 50 μM santamarine was used to treat HeLa cells for a specified time (inset), and the caspase 3 activity in the cell extract was detected by colorimetric analysis. The data was expressed as a percentage of the control sample. **p < 0.01 vs. the control group in **(C)** and **(D)**.

Escaping from apoptosis due to the intricate interaction of genetic aberrations and signaling pathways is one of the hallmarks of malignant cells (Hanahan and Weinberg, 2011). Activation of the apoptotic pathway in cancer cells is thus essential for cancer treatment. We herein perform a variety of assay methods to prove that the cytotoxicity of santamarine to HeLa cells is basically achieved by inducing cell apoptosis (Figure 6). Hoechst 33342 staining was initially used to evaluate chromatin condensation inducing apoptosis after santamarine treatment. As displayed in Figure 6A, the nuclei of untreated cells were stained weak blue, while bright chromatin condensation and nuclear fragmentation were observed in santamarine-treated cells. In addition, to evaluate the relative contribution of apoptosis to santamarine-induced cell death, we performed flow cytometry to analyze Annexin V-FITC and PI staining cancer cells. As shown in Figure 6B, it visually showed that the percentages of apoptotic cells increase following santamarine treatment for 24 h, especially at 50 μM. The statistical values from three independent experiments were also shown in Figure 6C. Both perfectly illustrated that santamarine induces cell apoptosis as the primary form of its cytotoxicity. Activation of caspase 3 is a crucial event in the process of cell apoptosis (Porter and Janicke, 1999). We thus finally tested the intracellular caspase 3 activity after santamarine treatment. HeLa cells were treated with santamarine (0–50 μM) for 24 h, or with 50 μM santamarine for 12 h or 24 h, and caspase 3 specific substrate Ac-DEVD-pNA was used to measure caspase 3 activity. Our results showed that santamarine significantly increased caspase 3 activity in HeLa cells in a dose- and time-dependent manner, indicating that HeLa cells undergo apoptosis after treatment (Figure 6D). These results collectively indicate that santamarine shows a pattern of inducing apoptosis in HeLa cells.

## Discussion

**FIGURE 7:**
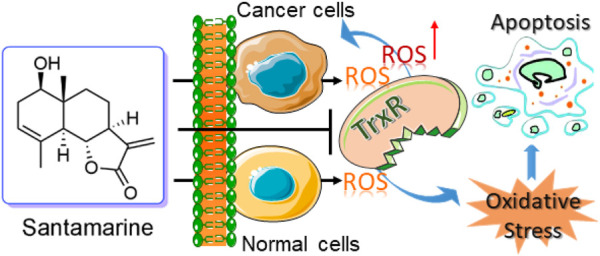
Revealing a new action mechanism of santamarine for targeting TrxR to induce oxidative stress-mediated apoptosis to selectively act on tumor cells.

Cancer cells proliferate and survive in the microenvironment because of the out-of-control of cell energetics as one of its hallmark events (Hanahan and Weinberg, 2011). As a result of cell energetics imbalance, cancer cells generate higher levels of ROS, accompanied by changes in antioxidant enzymes (Harris and Denicola, 2020; Zhang et al., 2021b). If the levels of ROS exceed the antioxidant capacity of these cells, it leads to excessive ROS accumulation, thereby inducing oxidative damage to the cells (Harris and Denicola, 2020; Zhang et al., 2021b). Therefore, cancer cells rely heavily on antioxidants including the thioredoxin system to maintain redox homeostasis to survive (Harris and Denicola, 2020; Zhang et al., 2021b). Although cancer cells and normal cells rely on antioxidant systems to maintain a reduced cell state, compared with normal cells, these cells have a narrower margin when reaching the maximum cytotoxicity threshold (Trachootham et al., 2009; Gorrini et al., 2013; Sabharwal and Schumacker, 2014). The sensitivity of ROS damage-related cell death becomes more obvious to adapt reasonably towards anticancer drug therapy (Zhang et al., 2021b). Accordingly, targeting the thioredoxin system is an effective way to eradicate cancer cells without causing other collateral damage to surrounding normal cells (Zhang et al., 2017b; Bian et al., 2019; Ghareeb and Metanis, 2020; Jastrzab and Skrzydlewska, 2021). The anticancer mechanism of santamarine targeting TrxR that we report here is a typical paradigm for this hallmark event of cancer cells (Figure 7).

As one of the crucial active molecules in *Inula racemosa*, it is reported that santamarine has fine antitumor activity and induces oxidative stress to suppress the activation of transcription factors NF-κB or STAT3 (Mehmood et al., 2017; Wu et al., 2017). However, how oxidative stress was induced is unclear. We demonstrated that santamarine targets TrxR to induce oxidative stress, thus providing a mechanistic explanation for the previous observations. Santamarine containing α-methylene-γ-lactone moiety (Figure 1) not only inhibited recombinant TrxR but also repressed cellular TrxR activity. Notably, santamarine harbors weak inhibitory activity on U498C TrxR and GR (Figure 3). This needs to be explained from the structural features and active centers of TrxR. Mammalian TrxR is a selenoprotein with a similar structure and catalytic mechanism to other pyridine nucleotide disulfide oxidoreductases (such as GR) (Zhong et al., 2000; Zhong and Holmgren, 2000; Gromer et al., 2003). Structurally, the mammalian TrxR holds an additional 16 amino acids with an exclusive -Gly-Cys-Sec-Gly catalytic motif, which forms the C-terminal redox-active center (Gromer et al., 2003). At physiological pH, the selenol group (SeH) in the penultimate C-terminal Sec residue in NADPH-reduced mammalian TrxR is completely ionized to the anionic form of selenate (Se^−^) (Byun and Kang, 2011). The high reactivity of nucleophilic selenate is easily attacked by electrophiles and alkylating agents, resulting in preferential inhibition of enzyme activity by santamarine. Santamarine not only inhibited the activity of TrxR in the cell, but also stimulated the ROS in HeLa cells, and further increased to reach the toxicity threshold, reduced the total sulfhydryl level, and finally induced oxidative stress (Figures 4, 5). Santamarine inhibits intracellular TrxR and stimulates robust ROS accumulation against HeLa cells. As a result, it finally overwhelms the stress response of cancer cells, explaining the results of santamarine’s selective inhibition of cancer cell proliferation (Figure 2). The cytotoxicity of chemotherapy administration on normal cells in patients is the main limitation of therapy (Firczuk et al., 2020). Our results also demonstrated that harnessing the potential to target the oxidative stress response in cancer cells could overcome this limitation. This will provide patients with more treatment strategies to take advantage of cancer changes in the redox defense system to combat cancer.

One of the hallmarks of cancer is the inherent or acquired circumvention of apoptosis, *i.e.*, evading apoptosis, due to the intricate interaction of genetic aberrations and signal pathways (Hanahan and Weinberg, 2011). Evading apoptosis not only leads to cancerous transformation and tumor progression, but can also engender treatment resistance (Fernald and Kurokawa, 2013). Therefore, most current chemotherapeutic drug treatments primarily work by activating cell death pathways including apoptosis. We presented that santamarine targets TrxR to induce oxidative stress-mediated HeLa cell death mainly through apoptosis (Figure 6), thus enhancing the potential of santamarine in cancer treatment. Both the internal mitochondrial pathway and the external cell surface receptor pathway of apoptosis are naturally occurring processes through which cells are directed to programmed cell death (Carneiro and El-Deiry, 2020). Santamarine inhibited TrxR in the cell and weakened the availability of the reduced Trx, thereby restricting the cellular function of Trx. Reduced Trx serves as an electron donor for a variety of antioxidant enzymes or proteins in the cell (Arner and Holmgren, 2000; Zhang et al., 2021a). The limited availability of the reduced directly affected the function of the enzymes of the antioxidant defense system in the cell, and ultimately resulted in a significant decline in the antioxidant defense capacity of the cell. Therefore, inhibition of TrxR by santamarine likely prompts oxidative stress-induced intrinsic mitochondrial-dependent apoptosis (Simon et al., 2000; Carneiro and El-Deiry, 2020). The previously published results have also witnessed this conclusion (Mehmood et al., 2017; Wu et al., 2017).

## Conclusion

In conclusion, we unveiled TrxR as a novel target of santamarine, and unraveled that santamarine represses TrxR to cause the disorder of cell redox homeostasis, which is beneficial to the mechanism of oxidative stress-mediated apoptosis in HeLa cells. Santamarine targeting TrxR reveals its previously yet-unrecognized mechanism of antitumor and provides a basis for the further development of santamarine as a potential cancer therapeutic agent.

## Data Availability

The original contributions presented in the study are included in the article/Supplementary Material, further inquiries can be directed to the corresponding author.
