# Supplementary material for: Inhibition of Thioredoxin Reductase by Santamarine Conferring Anticancer Effect in HeLa Cells
Source: Front Mol Biosci. 2021 Aug 18;8:710676. doi: 10.3389/fmolb.2021.710676 (PMC8416462; doi:10.3389/fmolb.2021.710676)
Supplement: Supplementary file 1 [file DataSheet1.docx]

Supplementary Material

Inhibition of thioredoxin reductase by santamarine conferring anticancer effect in HeLa cells

Junmin Zhang, Qianhe Xu, Hong-Ying Yang, Minghao Yang, Jianguo Fang, Kun Gao*

School of Pharmacy, State Key Laboratory of Applied Organic Chemistry, and College of Chemistry and Chemical Engineering, Lanzhou University, Lanzhou, 730000, China.

*** Correspondence:**Kun Gao
[npchem@lzu.edu.cn](mailto:npchem@lzu.edu.cn)


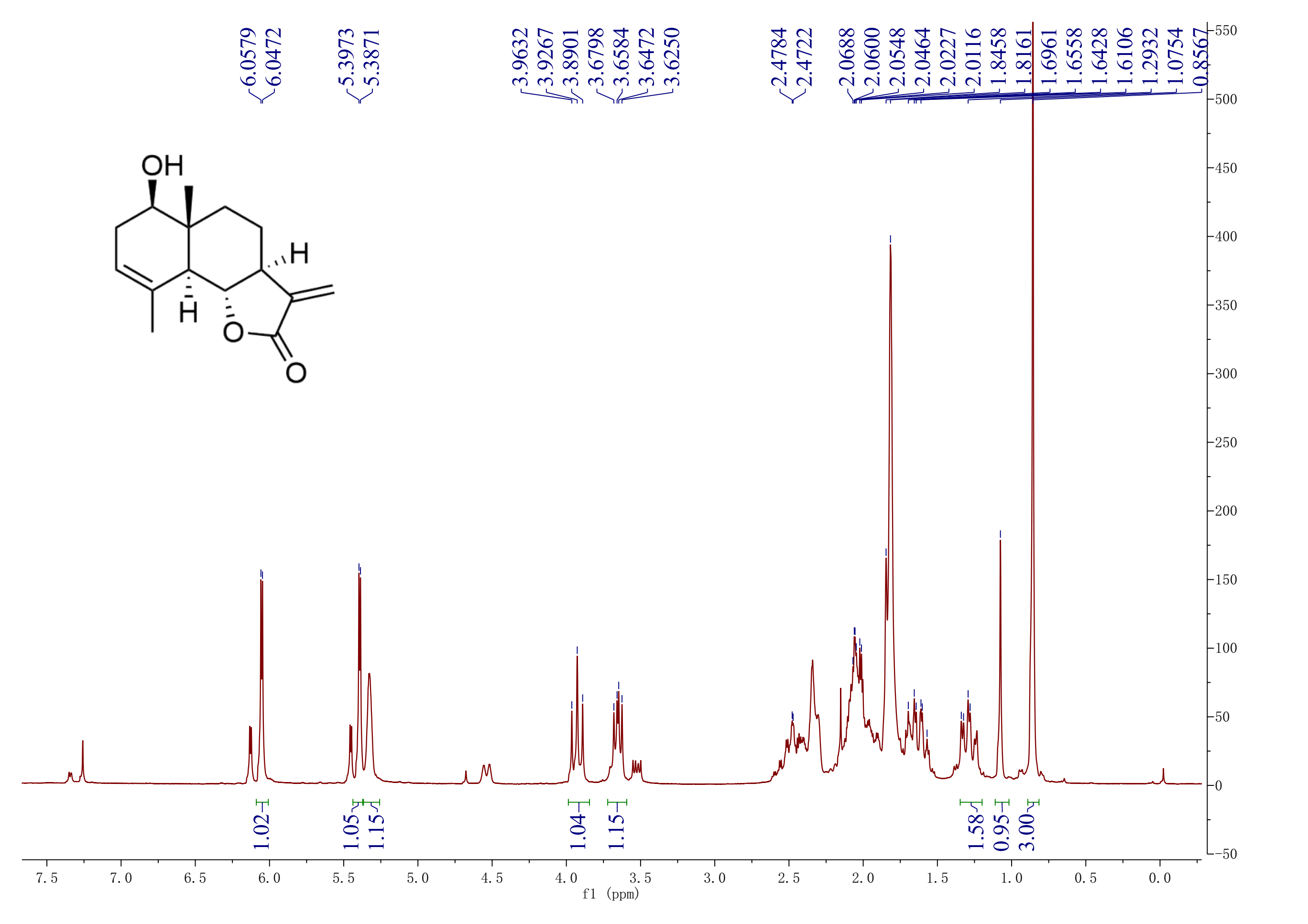


**Figure S1** ^1^H NMR spectrum of santamarine.


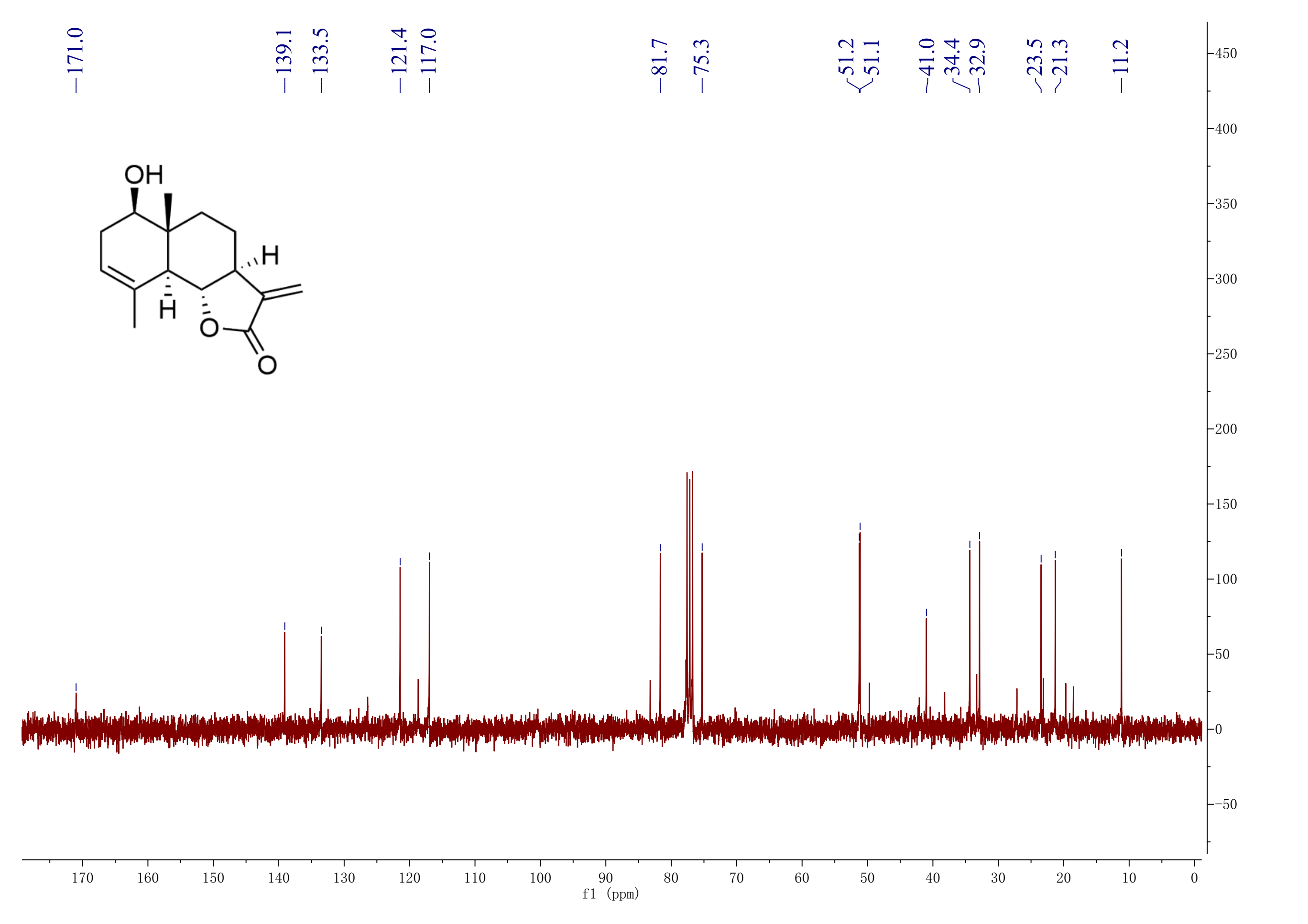


**Figure S2** ^13^C NMR spectrum of santamarine.


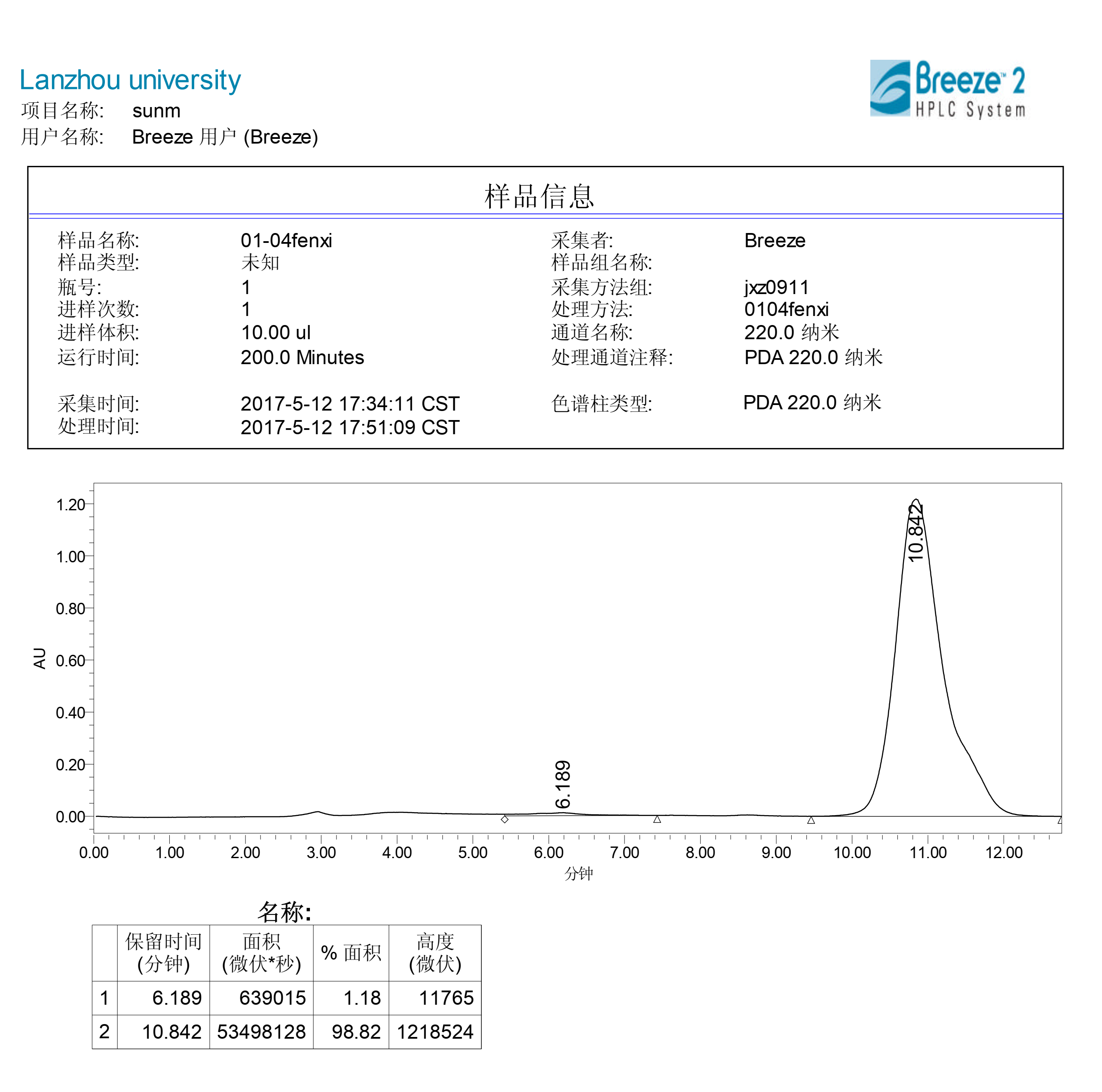
**Figure S3** Purity analysis of santamarine by HPLC.
